# Red Blood Cell Distribution Width Predicts Pulmonary Hypertension Secondary to Chronic Obstructive Pulmonary Disease

**DOI:** 10.1155/2019/3853454

**Published:** 2019-07-11

**Authors:** Jie Yang, Chuanmei Liu, Lingling Li, Xiongwen Tu, Zhiwei Lu

**Affiliations:** Department of Respiratory and Critical Care Medicine, Yijishan Hospital of Wannan Medical College, Wuhu City, Anhui Province, China

## Abstract

**Purpose:**

This study aims at investigating the predictive value of red blood cell distribution width (RDW) in pulmonary hypertension (PH) secondary to chronic obstructive pulmonary disease (COPD).

**Methods:**

213 eligible in-hospital COPD patients were reviewed between May 2016 and May 2018, including 39 cases with PH and 174 without PH. Clinical data including demographic characteristics, comorbidities, and results of ultrasound scans, imaging examinations, and laboratory tests were recorded.

**Results:**

Increased RDW level was observed in COPD patients with PH compared with COPD patients without PH, with 15.10 ± 1.72% versus 13.70 ± 1.03%, respectively (*p* < 0.001). RDW shared positive relationships with brain natriuretic peptide (BNP) (*p*=0.001, *r* = 0.513), pulmonary artery (PA) systolic pressure (*p*=0.014, *r* = 0.390), and PA-to-ascending aorta (A) ratio (PA : A) (*p*=0.001, *r* = 0.502). Multivariate analysis indicated that RDW, BNP, and PA : A > 1 were the independent risk factors of PH secondary to COPD (*p* < 0.05). The AUC of the RDW in patients with PH was 0.749 ± 0.054 (*p* < 0.001). The optimal cutoff value of RDW for predicting PH was 14.65, with a sensitivity and a specificity value of 69.2% and 82.8%, respectively.

**Conclusion:**

RDW is significantly increased in COPD patients with PH and thus may be a useful biomarker for PH secondary to COPD.

## 1. Introduction

Chronic obstructive pulmonary disease (COPD) is the fourth leading cause of death in the world; COPD is usually caused by exposure to noxious particles or gases and characterized by persistent airflow limitation and respiratory symptoms. [1] Pulmonary hypertension (PH) is one of the major complications of COPD and considered an independent prognostic factor for patients with COPD [2, 3].

Red blood cell distribution width (RDW) is a parameter for evaluating the variability of the circulating erythrocyte volume. An elevated RDW implicates dysfunctional erythropoiesis, increased red blood cell destruction, or shortened red blood cell lifespan [4]. RDW shares a close relationship with the prognosis of several diseases. Increasing data have indicated that RDW may be a promising predictor of the clinical outcome of cardiovascular or respiratory diseases, such as heart failure [5], PH [6], acute myocardial infarction [7], community-acquired pneumonia (CAP) [8], pulmonary embolism [9], and COPD [10, 11]. RDW is also a predictor of the mortality of COPD and pulmonary arterial hypertension patients [6, 11, 12]. However, the prediction value of RDW in PH secondary to COPD patients is unclear. Thus, we evaluate their association in this study.

## 2. Methods

### 2.1. Subjects

Consecutive AECOPD patients who were admitted to our hospital between May 2016 and May 2018 were reviewed in this retrospective study. The diagnosis of AECOPD is according to the Global Initiative for Chronic Obstructive Lung Disease (GOLD) criteria [1], and the COPD categories were as follows: group A, low risk and few symptoms; group B, low risk and more symptoms; group C, high risk and few symptoms; and group D, high risk and more symptoms. PH was defined as pulmonary artery (PA) systolic pressure (PASP) > 50 mmHg using the echocardiography method [13]. The exclusion criteria were as follows: existence of severe left heart failure, pulmonary embolism, connective tissue disorders, cancer, sleep apnea syndrome, renal failure, or asthma, and hospital confinement (days) > 30 days or < 2 days.

### 2.2. Data Collection

**Figure 1:**
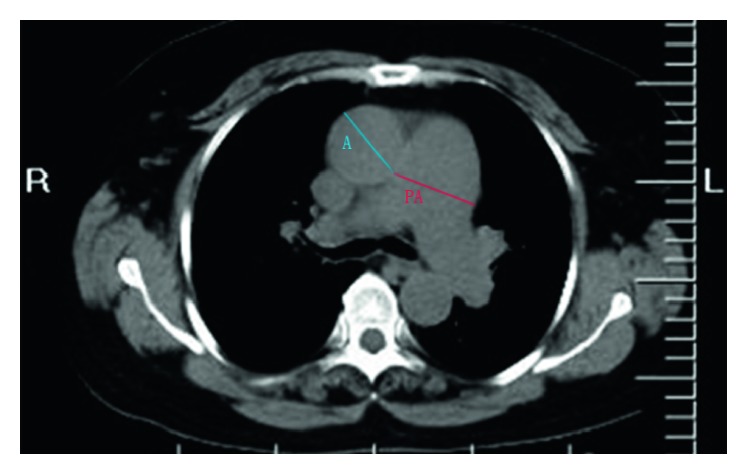
Measurement of PA and A diameters. PA, pulmonary artery; A, aorta. Diameters of PA and A were measured at the level of bifurcation.

Age, gender, body mass index (BMI), length of hospital confinement (days), systemic hypertension, diabetes, and coronary artery disease (CAD) were recorded. Laboratory parameters such as RDW (normal range of 11.5% to 14.5%), BNP, hemoglobin (Hb), white blood cell (WBC), neutrophils-to-leukocytes (N : L) ratio, C-reactive protein (CRP), and echocardiography, PA-to-aorta ratio (PA : A) were also collected; the diameters of PA and A were measured at the bifurcation level (Figure 1), and all data were obtained at admission.

### 2.3. Statistical Analysis

The software SPSS 19.0 (IBM Corp, Armonk, NY) was used for statistical analysis. A Kolmogorov–Smirnov test was adopted for the normality of distribution. Continuous variables were expressed as mean ± standard deviation (SD) and normally distributed. Categorical variables were expressed as percentage or number (*n*). Continuous data were analyzed through the independent-samples *t*-test, and categorical data were assessed by the chi-square test. The correlations between the RDW and the parametric variables were analyzed by the Pearson correlation method. Logistic regression analysis was used to reveal the independent risk factors of PH secondary to COPD. Receiver operating characteristic (ROC) curves were used to estimate the diagnostic value of RDW. A *p* value <0.05 indicated a significant difference, and the optimal cutoff value was based on the Youden index.

## 3. Results

213 eligible cases of patients with COPD and 39 cases with PH were enrolled in this study. The clinical characteristics are shown in Table 1. The age, BMI, length of hospital confinement, history of diabetes, hypertension, CAD, WBC, and N : L of the COPD patients with PH did not differ significantly from those of the COPD patients without PH. However, the COPD patients with PH had higher RDW levels, higher BNP levels, and increased PA : A compared with the COPD patients without PH (*p* < 0.05).

**Figure 2:**
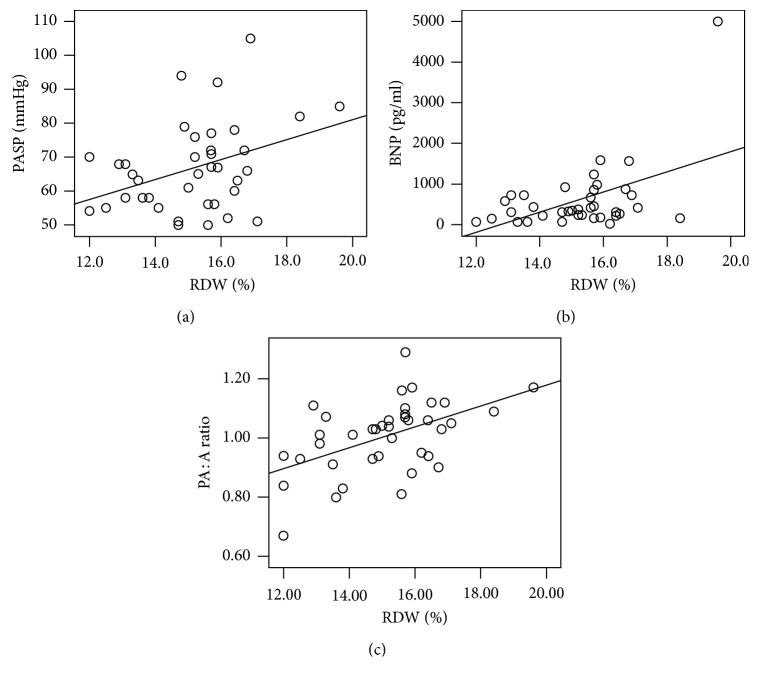
(a–c) Correlations of RDW levels with PASP, BNP, and PA : A. (a) RDW vs. PASP, *r* = 0.390, *R*^2^ = 0.152, *p*=0.014; (b) RDW vs. BNP, *r* = 0.513, *R*^2^ = 0.263, *p* < 0.001; (c) RDW vs. PA : A, *r* = 0.502, *R*^2^ = 0.270, *p* < 0.001. RDW, red blood cell distribution width; PASP, pulmonary artery systolic pressure; PA : A, pulmonary artery-to-ascending aorta ratio; BNP, brain natriuretic peptide.

According to the Pearson correlation analysis, the RDW shared positive correlations with PASP (*p*=0.014, *r* = 0.390), BNP (*p*=0.001, *r* = 0.513), and PA : A (*p*=0.001, *r* = 0.502), as shown in Figure 2.

**Figure 3:**
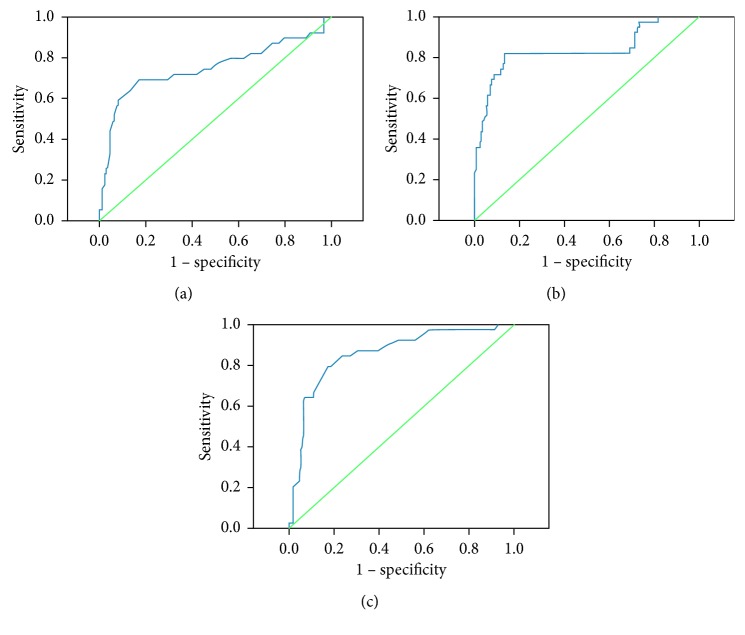
(a–c) ROC analysis for RDW, BNP, and PA : A predicting PH in COPD. (a) ROC curve with the RDW at identifying PH. The AUC was 0.749 ± 0.054 (*p* < 0.001). The optimal cutoff value of RDW for predicting PH was 14.65 and had a sensitivity and a specificity value of 69.2% and 82.8%, respectively. (b) ROC curve with the BNP level at identifying PH. The AUC was 0.837 ± 0.044. The optimal cutoff value of BNP for predicting PH was 146.105 and had a sensitivity and a specificity value of 82.1% and 86.8%, respectively. (c) ROC curve with PA : A at identifying PH. The AUC was 0.857 ± 0.035 (*p* < 0.001).The optimal cutoff value of PA : A for predicting PH was 0.925 and had a sensitivity and a specificity value of 79.5% and 82.8%, respectively. ROC, receiver operating characteristics; RDW, red blood cell distribution width; AUC, areas under the curve; BNP, brain natriuretic peptide; PH, pulmonary hypertension.

As shown in Table 2, multivariate analysis indicated that RDW, BNP, and PA : A were independent risk factors of PH secondary to COPD, with odds ratios of 1.521 (95% CI, 1.001–2.313; *p*=0.050), 1.007 (95% CI, 1.004–1.011; *p* < 0.001), and 5.365 (95% CI, 1.566–18.380; *p*=0.008), respectively. In Figure 3, the ROC curve analysis shows that the AUC value for RDW was 0.749 ± 0.054 (*p* < 0.001), BNP was 0.837 ± 0.044 (*p* < 0.001), and PA : A was 0.857 ± 0.035 (*p* < 0.001). The optimal cutoff values of RDW, BNP, and PA : A ratio for predicting PH were 14.65, 146.105, and 0.925, with sensitivity and specificity values of 69.2% and 82.8%, 82.1% and 86.8%, and 79.5% and 82.8%, respectively.

## 4. Discussion

This study evaluated the relationship between RDW and PH secondary to COPD. The main findings of this study were as follows. The COPD patients with PH had higher RDW levels than those without PH. RDW shared positive relationships with BNP, PASP, and PA : A. RDW, BNP, and PA : A > 1 were independent risk factors of PH secondary to COPD. Furthermore, the AUC of RDW for the diagnosis of PH in patients with COPD was 0.749 ± 0.054 (*p* < 0.001), with a sensitivity and a specificity value of 69.2% and 82.8%, respectively, when the optimal cutoff value of RDW was 14.65.

To date, several laboratory indexes have been applied in predicting the outcome of patients with PH, such as PA : A and BNP [14–17]. The latter is produced from the cardiac ventricle. BNP is known to be a useful index for diagnosing patients with left ventricular dysfunction, shares close relationships with right heart morphology and dysfunction, and parallels the extent of pulmonary hemodynamic changes and right heart failure [16, 18]. Additionally, BNP is a predictor of poor prognosis in primary PH. BNP concentrations show a close relation with mean pulmonary arterial hypertension. Consistent with previous studies, ours found that BNP level is correlated positively with pulmonary arterial pressure in patients with PH secondary to COPD.

PA enlargement is usually caused by resting PH and the centralization of blood flow caused by the destruction of the vascular bed. The PA : A measured by chest computed tomography (CT) scan is a potential predictor of PH and shares positive relationships with PA pressure in COPD patients [14, 19, 20] In addition, PA : A is dependently associated with acute exacerbations of COPD [21], and consistent with a previous study, ours found through CT that PA : A is correlated positively with pulmonary arterial pressure in patients with PH secondary to COPD.

However, the echocardiography is not the gold standard to diagnose PH, and there are several limitations such as insufficient accuracy and precision compared with invasive right heart catheterization (RHC), but with the technological improvements in echocardiography, it has increased its sensitivity for quantifying pulmonary artery pressure and it is now recognized as a safe and readily available alternative to right heart catheterization. Besides, invasive right heart catheterization has its own risks and complications [22, 23].

Accumulating data have revealed that elevated RDW level is associated with increased mortality in stable COPD patients [11]. RDW is independently associated with the prognosis of patients with PH (62% WHO category 1, 21% category 2, 3% category 3, 4% category 4, and 10% category 5) [6]. RDW can also predict survival in idiopathic pulmonary arterial hypertension patients [12]. Interestingly, we found that RDW is closely related to PA : A and BNP in COPD patients with PH, indicating that RDW may be a cost-effective alternative index for the PA : A scanned by chest CT and BNP. To identify the risk factors of PH in COPD patients further, logistic regression analysis showed that PA : A, RWD, and BNP contributed to the development of PH in COPD patients.

Increased RDW is usually due to ineffective red cell production and hemolysis; it is a novel predictor for mortality in many diseases, including CAP, acute stroke, chronic lung disease, and sepsis, especially in cardiovascular diseases, such as CAD, heart failure, and PH. RDW is closely related to BNP in patients with CAD and predicts the increased mortality of patients with CAD [24]. Furthermore, RDW could be a useful predictor for the severity of patients with chronic heart failure [25].

Notably, RDW is an independent risk factor of mortality and considered a better prognostic indicator than BNP in PH patients [6]. Moreover, RDW is related to the mortality of COPD patients [11]. In this study, we found that the RDW was positively correlated with PH in patients with COPD, which might be a useful predictor for the outcome of COPD patients with PH. and may be a cost-effective alternative to the more expensive BNP.

However, the mechanism underlying the increased RDW level in PH secondary to COPD is not well known. Previous studies found that a high RDW level is observed in cardiovascular or noncardiovascular diseases, such as poor pulmonary function, Down's syndrome, and dialysis, caused by oxidative stress and systemic inflammation [26, 27]. Hypoxemia is a risk factor of PH in COPD patients, that is, patients with PH secondary to COPD usually experience airflow obstruction and dyspnea which can aggravate oxidative stress. This condition is a major contributor to the development of COPD, indicating that oxidative stress might contribute to increased RDW levels in COPD patients with PH. However, the relationship between the degree of hypoxemia and RDW was not included in our study and hence requires further research.

Ineffective erythropoiesis due to chronic inflammation may be another contributor to the increased RDW level of COPD patients with PH. RDW is related to several inflammatory markers, such as CRP and ESR [27]. CRP is an acute phase protein secreted by the liver and is a biomarker for systemic inflammation in COPD. Other inflammatory cytokines, including tumor necrosis factor-*α*, interleukin (IL)-6, (IL)-8, and IL-1*β*, affect iron metabolism and bone marrow function; this situation inhibits erythropoiesis and the entry of larger juvenile cells into the peripheral circulation, thereby increasing the RDW [28, 29].

This study has several limitations. First, the sample size is small, especially the PH secondary to the COPD group. Second, RDW could be influenced by iron, folic, and vitamin B12, which were not appraised in our study. Certain inflammatory factors were also not assessed. Third, we did not perform sequential measurements of RDW. Finally, this was a retrospective study. All patients were AECOPD, and when the patients were admitted to our hospital, some were too severe to have lung function examined. So the association between lung function and RDW was not investigated, and this needs to be studied in future.

## 5. Conclusion

In the present study, we demonstrated that COPD patients with PH have higher RDW levels compared with those without PH. RDW may be a potential biomarker for the diagnosis of PH in COPD patients.

## Figures and Tables

**Table 1:** Baseline characters of demographic, clinical, laboratory tests, and CT.

Parameters	AECOPD with PH (*n* = 39)	AECOPD without PH (*n* = 174)	*p* value
Age, years (mean ± SD)	70.95 ± 6.36	70.95 ± 6.82	0.996
Males (*n*)	20	130	0.004
BMI (kg/m^2^)	20.4 ± 1.87	21.63 ± 3.60	0.133
COPD categories (*n*)			0.321
B	10	28	
C	2	15	
D	27	131	
RDW (%)	15.10 ± 1.72	13.70 ± 1.03	<0.001
WBC (10^3^/mm^3^)	6.74 ± 3.82	8.23 ± 4.65	0.065
Neutrophils (10^3^/mm^3^)	5.17 ± 3.33	6.58 ± 4.51	0.068
Leukocytes (10^3^/mm^3^)	1.01 ± 0.63	1.14 ± 0.60	0.209
N : L ratio	7.03 ± 7.25	7.65 ± 7.46	0.639
HB (g/L)	140.77 ± 21.46	129.10 ± 17.42	<0.001
PLT (10^3^/mm^3^)	148.15 ± 78.40	167.84 ± 66.43	0.107
Diabetes (*n*)	3	16	0.766
Hypertension (*n*)	13	74	0.291
CAD (*n*, %)	8	32	0.759
Hospital confinement (days)	9.10 ± 3.89	10.50 ± 11.36	0.452
BNP (pg/ml)	574.71 ± 839.00	113.00 ± 122.36	<0.001
PA : A ratio	0.99 ± 0.12	0.83 ± 0.12	<0.001

*Notes*. AECOPD, acute exacerbation of chronic obstructive pulmonary disease; PH, pulmonary hypertension; BMI, body mass index; COPD, chronic obstructive pulmonary disease; RDW, red blood cell distribution; WBC, white blood cell; N : L, neutrophils-to-leukocytes ratio; Hb, hemoglobin; PLT, platelet; CAD, coronary artery disease; BNP, brain natriuretic peptide; PA, pulmonary artery; A, aorta; PA : A, pulmonary artery-to-ascending aorta ratio.

**Table 2:** Independent risk factors for PH indicated by logistic regression model analysis.

Variable	Odds ratio	95% CI	*P*
RDW	1.521	1.001–2.313	0.050
BNP	1.007	1.004–1.011	<0.001
WBC	0.915	0.798–1.050	0.205
Sex (male)	0.814	0.279–2.372	0.706
Hb	1.023	0.995–1.051	0.103
PA : A ratio	5.365	1.566–18.380	0.008

RDW, red blood cell distribution width; BNP, brain natriuretic peptide; WBC, white blood cell; Hb, hemoglobin; PA : A, pulmonary artery-to-ascending aorta ratio.

## Data Availability

The data used to support the findings of this study are included within the article.
